# The Influence of Climate on Flourishing and Motivational Outcomes for U.S. Masters Swimmers

**DOI:** 10.3390/ijerph20031990

**Published:** 2023-01-21

**Authors:** Mary D. Fry, Troy O. Wineinger, Haiying Long, Marta Guivernau, Lori A. Gano-Overway, Susumu Iwasaki

**Affiliations:** 1 Department of Educational Psychology, University of Kansas, Lawrence, KS 66045, USA; 2 School of Foundations, Leadership and Administration, Kent State University, Kent, OH 44240, USA; 3 Department of Kinesiology, James Madison University, Harrisonburg, VA 22807, USA; 4 Department of Health and Human Performance, Fort Lewis College, Durango, CO 81301, USA

**Keywords:** caring climate, task-involving climate, older adults, effort, enjoyment, sport

## Abstract

The climate in which older adults exercise and participate in sport may play a role in promoting a lifetime commitment to exercising. However, little research has examined the relationship of caring (C) and task-involving (TI) climates, motivation, and well-being with respect to older adult athletes. The purpose of this study was to examine the relationship between Masters swimmers’ perceptions of the climate, effort, enjoyment, and flourishing as well as explore the mediating effects of effort and enjoyment on the relationship between climate and flourishing. U.S. Masters swimmers (*n* = 294; *M*_age_ = 63.57 years; 84.40% White) with 1–80 years of swimming experience (*M* = 34.54 years) participating in coach-led programs completed an online survey. The results of latent variable, multiple-mediator analyses via structural equation modeling revealed two important contributions to the literature: (1) when Masters swimmers perceived that they were in C and TI climates, they were more likely to report higher levels of effort and greater enjoyment and flourishing; (2) the Masters swimmers’ effort levels directly influenced their flourishing, mediating the relationship between climates and flourishing. This research has important implications for practice and policy, as U.S. Masters Swimming appears to be a fruitful avenue for promoting an enjoyable physical activity that can be experienced throughout a lifetime.

## 1. Introduction

Physical activity provides a multitude of health benefits that contribute to adaptive overall functioning for individuals of all ages, but especially for older adults [1]. Routine physical activity enhances physiological and psychological functioning, which, in turn, reduces the risk of falling; succumbing to cardiovascular disease, which is the foremost cause of death in the U.S.; and depression [2,3,4]. Physical activity also promotes fitness (e.g., strength, cardiovascular endurance, flexibility, and balance), which is important for optimal quality of life [5,6,7]. Despite the many benefits of physical activity, the majority of older adults are missing the opportunity to improve their health and quality of life due to their physically inactive lifestyles [8]. The United States Department of Health and Human Services [6] recently issued a report with guidelines for physical activity; for optimal health benefits, the guidelines suggest that adults should attain between 150 and 300 min of moderate-intensity physical activity per week. Additionally, they state that although there have been improvements in physical activity levels among adults, roughly 80 percent of American adults are not meeting guidelines. 

Apart from not meeting physical activity guidelines, more than one-third of adults who are 45 and older feel lonely, and nearly one-fourth of adults 65 and older are considered socially isolated, as reported by the National Academies of Sciences, Engineering, and Medicine [9]. This is alarming, as Lee et al. [10] found in their longitudinal research that, irrespective of other social experiences, higher levels of loneliness were associated with greater depression symptoms. They called for physical activity leaders to socially engage older adults in order to reduce their isolation and subsequent loneliness, which may help combat this growing public health concern. U.S. Masters Swimming is one organization that is exemplary with respect to this effort to offer social engagement for older adults to promote physical activity across one’s lifespan [11].

U.S. Masters Swimming is a nonprofit, national, member-operated organization that consists of over 1500 swimming clubs and workout groups, and it provides membership benefits to roughly 70,000 U.S. Masters swimmers across the country [11]. U.S. Masters Swimming offers programs and teams for all skill levels to encourage participation, as well as coaches who will provide personalized workouts, feedback, and instruction. Brilliant et al. [12] investigated U.S. Masters swimmers’ motivation for swimming in their later years and found that enjoyment, slowing the decline of their aging bodies, increased competence, team structure, and affinity for the sport were major contributing factors in their desire to continuing to swim. Research conducted with Masters swimmers at the 2008 World Championship also highlighted enjoyment as being an antecedent correlate of one’s commitment to being physically active [13]. Therefore, effort and enjoyment appear to be key variables linked to sustaining motivation and having optimal experiences in physical activities such as swimming. These findings are noteworthy, as swimming has been shown to be an adaptive form of exercise for older adults since it is non-weight bearing, safe (i.e., offering a reduced risk of falling), and effective in terms of energy consumption [14,15]. Given that swimming is a beneficial form of physical activity throughout a lifespan, U.S. Masters Swimming has been fervently promoting swimming to older adults [11]. Although the health benefits of being physically active are prevalent and organizations such as U.S. Masters Swimming are providing opportunities in this regard, there is a continuing need to explore and better understand how adults’ physical activity-related experiences are structured in order to promote motivation and well-being.

Motivation is clearly a major contributing factor to older adults’ experiences and long-term participation in physical activity [12,16]. One theory that is particularly salient with respect to optimizing motivation is achievement goal perspective theory [17,18,19]. The researchers behind this theory maintained that fostering a task-involving (TI) climate, wherein athletes gauge their success based on personal effort and improvement rather than their normative standing among competitors, would improve motivational outcomes. Nicholls [18,19] and other motivational researchers [20,21,22] identified additional features of this climate, which include encouraging cooperation among teammates, striving to allow all participants to see how they play an important role on the team, and viewing mistakes as part of the learning process. Developing a TI climate could be very appealing for Masters swimmers as they seek to focus on controllable factors associated with their effort and personal development in the midst of the aging process.

Newton et al. [23] added a caring dimension to the TI features, whereby they defined a caring (C) climate as “the extent to which individuals perceive a particular setting to be interpersonally inviting safe, supportive, and capable of providing the experience of being valued and respected” (p. 70). A recent review of the C climate research demonstrated positive associations between caring, social behaviors, psychological well-being, and motivational outcomes [24]. This type of environment may be particularly important for older adults, as they may especially benefit from perceiving they are in an inviting and supportive community where they may develop social ties to support their well-being and motivation to be more physically active.

Although these climates are predicted to benefit older adults, research investigating their perceptions of the climate and associated outcomes is currently lacking. However, research has consistently found benefits for athletes of younger ages and across sport contexts with respect to perceiving a C and TI climate, including enhanced effort, enjoyment, and indicators of flourishing [25,26,27,28]. Specific to the college exercise setting, Brown and Fry [29] found that exercisers’ perceptions of a C and TI climate in their aerobic classes were positively associated with their reporting higher levels of intrinsic motivation, effort, and enjoyment. Additionally, Huddleston et al. [30] found that employees’ perceptions of a motivational climate in corporate fitness centers were associated with known correlates of intrinsic motivation (i.e., effort/improvement, interest/enjoyment, and perceived competence). Of particular interest in this study is how C and TI climates may influence Masters swimmers’ motivational responses in their endeavors of being physically active. 

Another vital area of research to explore among older athletes is the link between the C and TI climates and aspects of well-being. Previous research has consistently associated C and TI climates with parameters of well-being (e.g., coping appraisals, cognitive stress levels, hope, happiness, and self-kindness [28,31]). However, the level of flourishing has not been investigated. Keyes [32,33] defined flourishing as a state of complete mental health wherein individuals are free from mental illness and have a high level of subjective well-being with an optimal level of psychological and social functioning. A link has been made between flourishing and physical activity. In their work, O’Rourke et al. [34] found that flourishing likely supports the achievement of guidelines for physical activity for adults. When surveying a group of older adults (*M*_age_ = 62 years), Leibow et al. [35] reported that flourishing worked indirectly through a positivity ratio (i.e., ratio of positive to negative emotions) to predict a later increase in exercise over the course of 3 to 5 years. Additionally, they found that initial exercise frequency was associated with later improved flourishing and positivity ratio scores over the same period. Thus, the current work suggests that in addition to motivational variables, such as effort and enjoyment, flourishing may be a key factor influencing the enhancement of physical activity. Further, researchers observed that athletes who reported flourishing also indicated that they were in a supportive sport environment [36], thereby suggesting that perceptions of C and TI climates may be positively linked to flourishing. 

Overall, current research among younger athletes supports relationships between C and TI climates, effort, and enjoyment, yet limited work has been conducted with older populations. Further, current work suggests a potential link between C and TI climates and flourishing. Therefore, the primary purpose of this study was to examine the relationship between Masters swimmers’ perceptions of climate, effort, enjoyment, and flourishing. It was hypothesized that swimmers who reported perceiving high C and TI climates were expected to be more likely to report higher effort, enjoyment, and flourishing.

Some recent studies also found direct relationships between flourishing and indicators of effort and enjoyment. For example, in his work assessing Masters athletes, Salama-Younes [37] found that there was a significant negative correlation between athletes’ flourishing and their psychological distress. Further, Padhy et al. [38] reported positive relationships between flourishing, subjective vitality, and the one-grit subscale, i.e., the perseverance of effort among a wide age range of adults. This latter finding, that is, the positive association between flourishing and perseverance of effort, has also been reported in college-age students across multiple cultures [39]. Therefore, the secondary purpose of this study was to explore whether effort and enjoyment may mediate the relationship between C and TI climates and flourishing. Given that this area has not been previously explored, no direct hypotheses were set.

## 2. Method

### 2.1. Participants

U.S. Masters swimmers (*n* = 259; female 55.40%) were invited to complete a brief survey in which they provided feedback on their swimming experiences in the organization. They ranged in age from 55 to 87 years (*M_age_* = 63.57, *SD* = 7.19) and in terms of years of experience (1–80 years; *M* = 34.54, *SD* = 21.67). In addition, swimmers reported being White (84.40%), Hispanic/Latino (1.70%), and other/missing (13.90%).

### 2.2. Procedure

U.S. Masters Swimming administrators distributed a link to an anonymous Qualtrics survey (and an invitation to participate in the study) to all members in their database. The survey remained open for six weeks. The Institutional Review Board at the first author’s university approved this study, and informed consent was obtained from all participants through the survey.

### 2.3. Measures

The survey consisted of the following measures: swimmers’ perceptions of the climate (i.e., C and TI); their personal effort, enjoyment, and sense of flourishing in their time with the Master’s swimming program; and demographic information (e.g., age, race, years of swimming experience, etc.).

### 2.4. Caring Climate

The 13-item Caring Climate Scale (CCS; Newton et al. [23]) was used to assess the extent to which swimmers perceived their psychosocial environment to be interpersonally inviting, safe, and a place where all are treated with kindness and respect. The stem for the items is “At my swim facility…”, and a sample item is “coaches care about the swimmers.” Swimmers responded on a 5-point Likert scale ranging from 1 (“*Strongly Disagree*”) to 5 (“*Strongly Agree*”). Newton et al. [23] provided support for the internal reliability (α = 0.92) and validity of the CCS with physical activity participants. In the current study, Cronbach’s alpha and composite reliability for all the items measuring CC are 0.94 and 0.89, respectively. All items of this measure are negatively skewed, with the skewness ranging between −2.87 and −1.09. They all have positive kurtosis, and some have very high kurtosis (e.g., items 1, 2, and 3).

### 2.5. Perceived Motivational Climate

Swimmers’ perceptions of the extent to which they perceived their swimming environment to embody TI motivational climate features was assessed with the 6-item TI climate scale of the Perceived Motivational Climate in Exercise Questionnaire—Abbreviated [40]. The stem for the items was “At my training facility…” and a sample item is “coaches encourage swimmers to help each other learn.” Swimmers responded to the items using a 5-point Likert scale ranging from 1 (“*Strongly Disagree*”) to 5 (“*Strongly Agree*”). Moore et al. [40] conducted Confirmatory Factor Analyses with physical activity participants and reported the measure to be satisfactory for use in exercise settings. Additionally, they found the measure to display acceptable reliability (α = 0.77–0.79) across three respective studies. In the current study, Cronbach’s alpha and composite reliability for all the items measuring TI climate are both 0.83. All items of this measure are slightly negatively skewed. All but one item have positive kurtosis.

### 2.6. Effort

The 4-item effort subscale of the Intrinsic Motivation Inventory (IMI; McAuley et al. [41]) was employed to assess swimmers’ perceptions of their exerted effort. Swimmers responded using a 5-point Likert scale, with responses ranging from 1 (“*Strongly Disagree*”) to 5 (“*Strongly Agree*”). A sample item included, “I put a lot of effort into swimming.” This measure has demonstrated acceptable validity and reliability (α = 0.84) in the physical activity setting [41]. In the current study, Cronbach’s alpha and composite reliability for all the items measuring effort are 0.70 and 0.68, respectively. Three items of this measure are slightly negatively skewed, and one is slightly positively skewed. One item has negative kurtosis, while the others have positive kurtosis. 

### 2.7. Enjoyment

Swimmers’ perceptions of enjoyment were assessed using the 5-item enjoyment subscale of the IMI [41]. Swimmers responded to items using a 5-point Likert scale, with responses ranging from 1 (“*Strongly Disagree*”) to 5 (“*Strongly Agree*”). A sample item is as follows: “I enjoy swimming.” McAuley et al. [41] reported acceptable validity and reliability (α = 0.78) for the scale. In the current study, Cronbach’s alpha and composite reliability for all the items measuring enjoyment are 0.72 and 0.71, respectively. All but one item is slightly negatively skewed. All items have positive kurtosis, with item 1 having very high kurtosis.

### 2.8. Flourishing

The 8-item Flourishing Scale [42] was utilized to assess swimmers’ perceptions of personal flourishing in their lives. Swimmers responded using a 5-point Likert scale, with responses ranging from 1 (“*Strongly Disagree*”) to 7 (“*Strongly Agree*”). A sample item is, “I actively contribute to the happiness and well-being of others.” This measure has demonstrated acceptable validity and reliability (α = 0.82) with a sample of older participants (*M_age_* = 66; Fassih-Ramandi et al. [43]). In the current study, Cronbach’s alpha and composite reliability for all the items measuring flourishing are 0.90 and 0.89, respectively. All items of this measure are slightly negatively skewed. They have positive kurtosis, and item 1 has high degree of kurtosis.

### 2.9. Data Analysis

To examine the relationships among the variables, we employed latent variable multiple-mediator mediation analyses through a Structural Equation-Modeling (SEM) approach, which allows for the examination of both measurement and structural models simultaneously [44] and provides an unbiased estimate of multiple mediation effects [45]. A Confirmatory Factor Analysis (CFA) was performed before the SEM mediation analyses to ensure sufficient construct validity of the measurement model [46]. To interpret the results of CFA and SEM, we used model fit indices of Comparative Fit Index (CFI), Tucker–Lewis Index (TLI), Root-Mean-Square Error of Approximation (RMSEA), and Standardized Root-Mean-Square Residual (SRMR). A model is often considered a good fit with the data if CFI and TLI are 0.90 or above, RMSEA is 0.06 or below, and SRMR is 0.08 or below [47,48].

Preliminary determinations of data quality and missingness were assessed in IBM SPSS version 28 [49] and through the examination of descriptive statistics and correlations. For example, we checked the normality of the items by using their skewness and kurtosis values, Kolmogorov–Smirnov test [50], and normal probability plots. All results suggested violation of the normality assumption. Linearity and homoscedasticity of the data were also checked by using score and residual scatterplots [51]. Linearity of the relationships was supported, but the residuals were heteroscedastic. The main analyses of CFA and SEM were all conducted using the *lavaan* package within R environment [52]. We used an estimator of MLR to account for the non-normal and heteroscedastic data. We found 8% of missing values in all observations (*n* = 21). Little’s MCAR (Missing Completely At Random) test yielded a significant result (χ^2^_407_= 534.53, *p* < 0.001), suggesting that the missing values were either missing at random or non-ignorable. To account for the missing data, full information maximum likelihood (FIML) was implemented due to its superior performance over other approaches to handling different types of missing values [53]. The *standardized* function was used to obtain all standardized coefficients of the variables. Many methods have been suggested for testing mediated or indirect effects over the past few decades. We selected the bootstrap CI method (with 1000 bootstraps), which randomly resamples with replacements to estimate effect size and confidence interval of the population. It is advantageous compared to other approaches because it can control for Type I errors while yielding an accurate value [54,55].

## 3. Results

### 3.1. Descriptive Statistics and Correlations

The majority of the swimmers reported perceiving the climate in their swimming contexts as moderately C and TI. In addition, most swimmers reported moderate levels of effort and enjoyment in their sport and flourishing in life (see Table 1). 

The Pearson’s correlations for all the constructs were significant at the *p* < 0.001 level, except for the correlation between flourishing and the C climate, which was significant at the *p* < 0.05 level (see Table 2).

### 3.2. Validity of the Measurement Model

**Figure 1:**
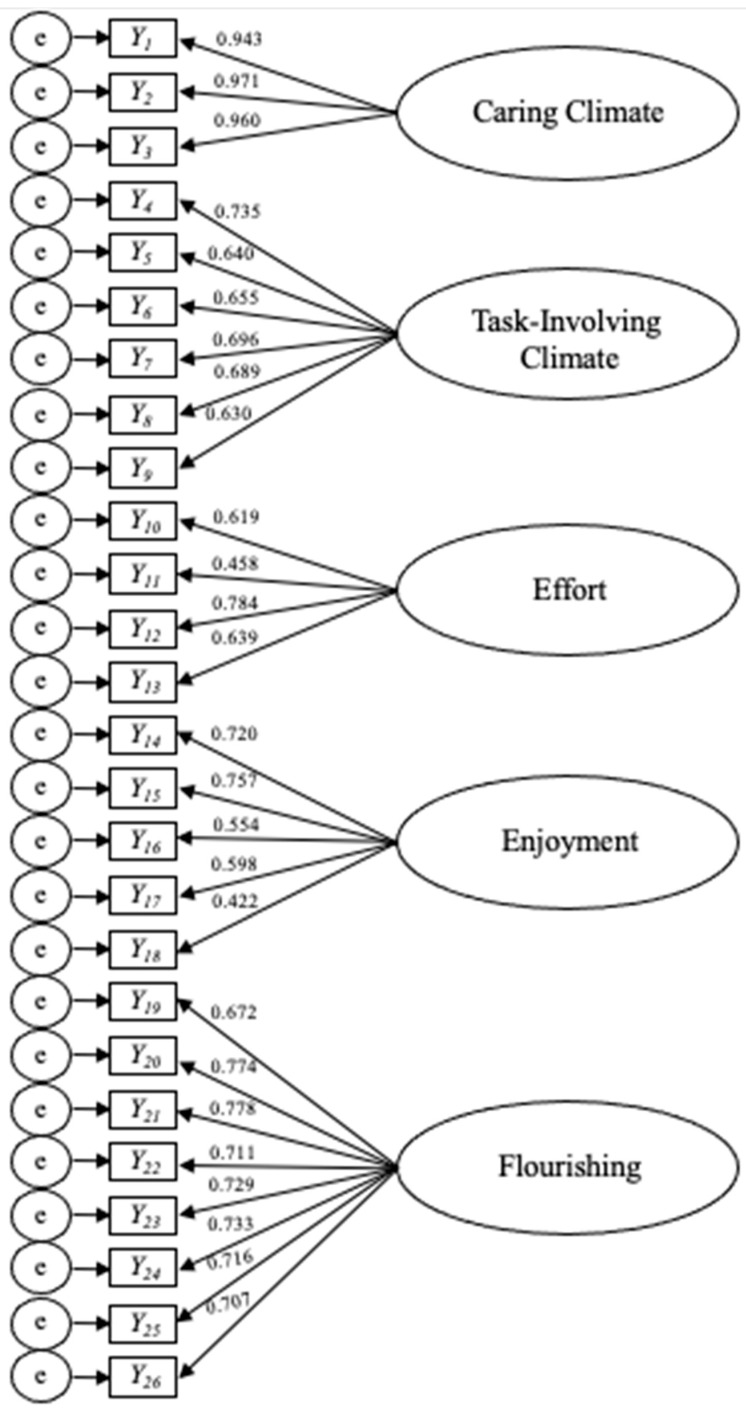
Final Measurement Model. Note: Model fit (CFI = 0.93, TLI = 0.92, RMSEA = 0.06, and SRMR = 0.05). All factor loadings are significant at *p* < 0.001.

We first examined the construct validity of all the measures by using CFA with all the items that measure the latent variables. The fit indices were not adequate (CFI = 0.77, TLI = 0.75, RMSEA = 0.09, and SRMR = 0.10). A few items were found to have low factor loadings with respect to the latent variable of the C climate (e.g., item 10′s factor loading was only 0.38). This was not surprising, as previous studies encountered the same issue (e.g., Wineinger et al. [56]). To address this issue, the item-parceling approach [57] was employed, in which a subset of items measuring a C climate was initially grouped into three parcels (i.e., Y1, Y2, and Y3; see Figure 1) and were used to measure the latent variables in all later analyses. This approach has been widely applied in CFA and SEM [58,59], and researchers believe it can reduce the complexity of the statistical model, require a smaller sample size, and may solve distribution non-normality issues [57,59]. After item parceling was performed for the C climate, the fit indices of the measurement model significantly improved and indicated a good fit with the data: CFI = 0.93; TLI = 0.92; RMSEA = 0.06; SRMR = 0.05. The ratio of the chi-square and the degree of freedom in the model was 1.92, which was also indicative of a good model fit. The three parceled subscales had very strong factor loadings with respect to the variable of a C climate (λ = 0.94, 0.97, and 0.96). The factor loadings of all the items measuring other latent variables primarily fell between 0.60 and 0.78 (see Figure 1). The Average Extracted Variance (AEV) for the five latent variables was 0.92, 0.45, 0.38, 0.35, and 0.53, indicating that the C climate and flourishing measures had better convergent validity than the other three variables. The square roots of each AEV were 0.96, 0.67, 0.62, 0.59, and 0.73, which were greater than all the correlations with the other latent variables and indicated great discriminant validity [60].

### 3.3. Mediation Analyses

**Figure 2:**
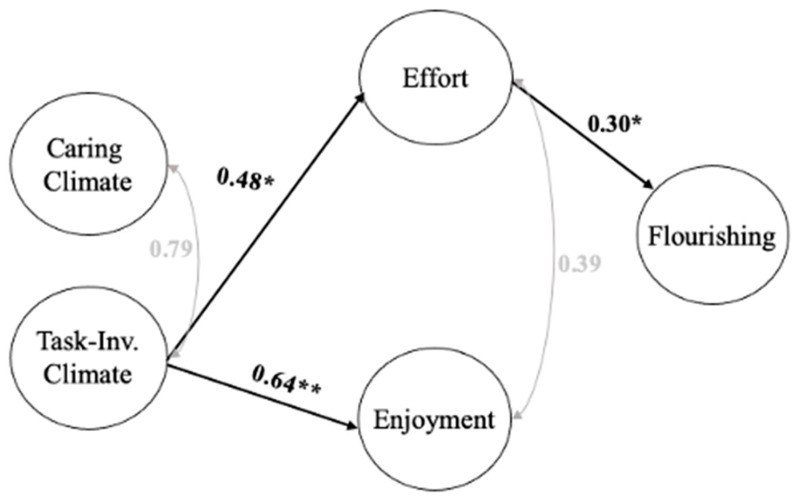
Mediation Analyses: Model 1. Note: Model fit (CFI = 0.93, TLI = 0.92, RMSEA = 0.06, and SRMR = 0.05). Significant theory-driven regression paths (* *p* ≤ 0.05; ** *p* ≤ 0.001) are presented in the model. Standardized regression coefficients are presented for each significant path. The significant indirect effect was as follows: Task-Involving Climate → Effort → Flourishing (0.14).

First, we performed a mediation analysis, with two climate variables (i.e., C and TI) used as the exogenous variables; effort and enjoyment being two mediators; and flourishing selected as the endogenous variable (Model 1; see Figure 2). Both the C and TI climates did not have a significant direct effect on flourishing (see Table 3). The C climate did not significantly predict effort or enjoyment, but the TI climate did. Enjoyment was not a significant predictor of flourishing, while effort was. Further, the C climate did not have a significant indirect effect on flourishing through effort or enjoyment, and while the TI climate did not have a significant indirect effect on flourishing through enjoyment, it had a significant indirect effect on flourishing through effort. The non-significant direct and indirect effects of the C climate may be due to its high correlation with the TI climate (*r* = 0.79). Researchers [56,61] have reported the same statistical idiosyncrasy (i.e., a high degree of correlation between the C and TI climate scales likely prevents the C climate construct from making a unique contribution to the analyses). Thus, we performed two separate mediation analyses with each climate as the only exogenous variable in the model. 

**Figure 3:**
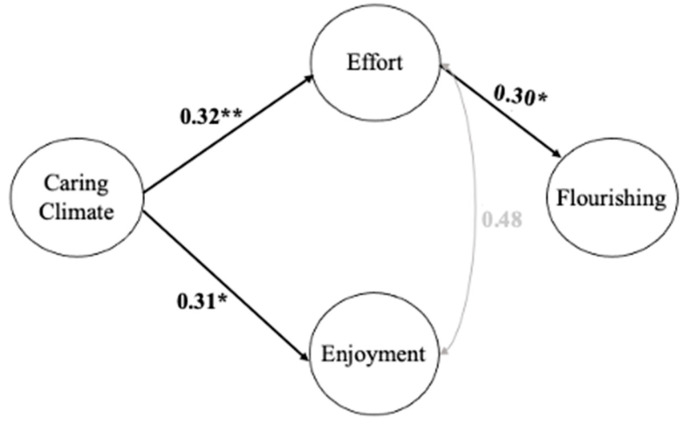
Mediation Analyses: Model 2. Note: Model fit (CFI = 0.93, TLI = 0.92, RMSEA = 0.06, and SRMR = 0.06). Significant theory-driven regression paths (* *p* ≤ 0.05; ** *p* ≤ 0.001) are presented in the model. The standardized regression coefficients are presented for each significant path. Significant indirect effect was: Caring Climate → Effort → Flourish (0.10).

For the model with the C climate as the exogenous variable, effort and enjoyment as the two mediators, and flourishing as the endogenous variable, the C climate did not have a significant direct effect on flourishing, but it significantly predicted both effort and enjoyment (Model 2, see Figure 3). In addition, effort had a significant direct effect on flourishing, whereas the effect of enjoyment on flourishing was not significant. The three significant direct effects were all above 0.30, or at a moderate level. Further, a significant indirect effect—at 0.10—was found regarding the effects of a C climate on flourishing through effort (see Table 4). 

**Figure 4:**
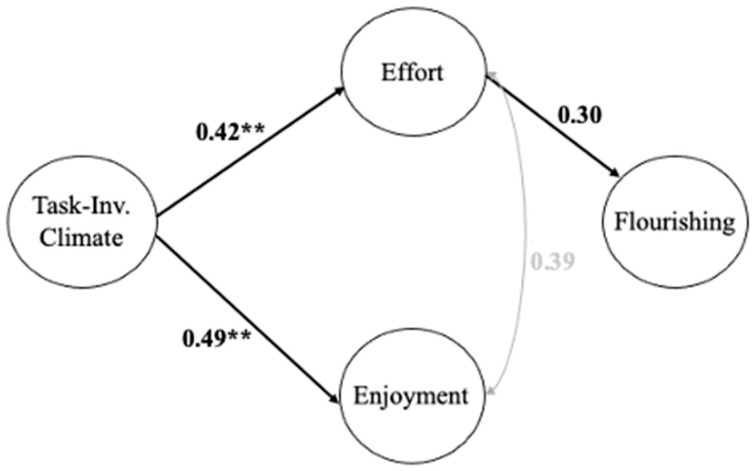
Mediation Analyses: Model 3. Note: Model fit (CFI = 0.90, TLI = 0.88, RMSEA = 0.06, and SRMR = 0.06). Significant theory-driven regression paths (** *p* ≤ 0.001) are presented in the model. The standardized regression coefficients are presented for each significant path. The significant indirect effect was: Task-Involving Climate → Effort → Flourishing (0.12).

The model with the TI climate as the exogenous variable, effort and enjoyment as two mediators, and flourishing as the endogenous variable shows that the TI climate did not have a significant direct effect on flourishing, but this climate had a significant effect on both effort and enjoyment (Model 3, see Figure 4). Similar to the C climate model, effort was found to have a significant effect on flourishing, while the effect of enjoyment on flourishing was non-significant. Further, a significant indirect effect was found regarding the effect of a TI climate on flourishing through effort. These patterns are consistent with the C climate model. However, the significant direct effects of the TI climate on the two mediators (effort and enjoyment) were above 0.40, which were slightly larger than those in the C climate model. The indirect effect of the TI climate on flourishing through effort (0.12) was close to that in the C climate model (0.10; see Table 5). 

## 4. Discussion

Older adults experience a myriad of benefits from engaging in physical activity, yet only a minority of adults meet the recommended physical activity guidelines [6]. Motivation and well-being play key roles in determining engagement in physical activity. Therefore, organizations such as U.S. Masters Swimming are interested in learning more about determining methods to enhance the motivation and well-being of their members to encourage physical activity. This study provides an initial exploration of methods for enhancing motivation and well-being by examining the relationship between Masters swimmers’ perceptions of the C and TI climates created within their swim teams, their motivational outcomes (i.e., effort and enjoyment in swimming), and their well-being (i.e., flourishing). 

The primary purpose of this study was to investigate the correlations between perceptions of climate, effort, enjoyment, and flourishing. The hypothesized relationships were supported. Specifically, Masters swimmers who perceived that their team climate consisted of focusing on applying effort to reach one’s potential and striving to improve were more likely to report greater effort, enjoyment, and flourishing. Further, when these older athletes perceived their swim team environments to be welcoming, respectful, kind, and safe, they reported a more enjoyable sport experience for which they wished to apply greater efforts and flourish. These findings logically align with achievement goal perspective theory [17,18,19] and the conceptual C climate model [24]. According to achievement goal theorists, being in an environment in which one is rewarded and evaluated based on effort and improvement leads to greater individual effort. Further, greater effort and enjoyment are postulated to occur as individuals are encouraged to monitor factors within their control and for which they can perceive progress. Exploring these findings from the lens of the C climate model, enjoyment and well-being may result when individuals are in an environment that supports their needs and interests and provides a sense of belonging. Further, a connection to perceived effort may result from reciprocity, which has been proposed as a potential mechanism explaining the relationship between perceptions of a C climate and athlete outcomes [24]. For example, athletes may have a desire to display reciprocal behavior towards a program that has provided a warm and inviting climate by working harder. Additionally, it may follow that when individuals perceive a C and TI climate, they feel valued and supported with respect to reaching their potential, which may result in enhanced confidence and, therefore, the greater application of effort. These forms of reciprocity have been explored in the relevant research, wherein former collegiate athletes discussed their experiences of gratitude toward their coach and how it led to enhanced motivation [62], as well as with a high school athlete feeling more confident when her coach believed in her potential, which caused her to remain committed to her sport [63].

Across ages and sport contexts, motivational researchers have also found that individuals will be more likely to report greater effort and enjoy their experience in C and TI climates [25,26,30,64]. Additionally, Santi et al. [65] found that Masters swimmers who reported having teammates and coaches with supportive and not expectant attitudes were more likely to be committed to their swimming. Further, Kuettel et al. [36] demonstrated that high-level performance athletes categorized as a having a flourishing mental health profile reported a more supportive team environment than those with a moderate or languishing mental health profile. Although this study does support and connect to previous work, it is unique in its establishment of these findings with a new population of older adult athletes. 

The secondary purpose of this study was to explore the mediating effects of effort and enjoyment between C and TI climates and flourishing. The full mediation model with both climate scales included showed that the TI climate could directly predict swimmers’ effort and enjoyment in swimming as well as indirectly effect flourishing via effort. Although the C climate did not appear to initially predict any of the outcome variables, this appears to be due to the high correlation between the C and TI climates (*r* = 0.79). However, an examination of the mediation model that was run separately for each climate found that the C climate could directly predict swimmers’ levels of effort and enjoyment. Additionally, in this model, effort was found to have a direct effect on swimmers’ flourishing while the effect of enjoyment was not significant. Comparable results were found when running the mediation analyses with only the TI climate. Testing the effects separately for the C and TI climate scales revealed that the lack of significance seen in the full mediation model with both climate scales was due to statistical idiosyncrasies and was not the result of the C climate having an insignificant contribution.

This study provides another unique contribution to the literature by noting the role of effort in mediating the connection between climate variables and flourishing. As described earlier, there is both conceptual and empirical evidence supporting C and TI climates’ links to effort. Further, the direct link between effort and flourishing supports previous research that found a relationship between flourishing and another indicator of effort. Specifically, scholars have demonstrated that the perseverance-of-effort subscale of the grit measure is positively associated with flourishing [38,39]. Describing this connection, Datu et al. [39] not only proposed a connection to conscientiousness but also contended that individuals who persevere and are grittier will seek out ways to achieve success and happiness. Thus, it may be that individuals who apply effort in their swimming may also apply this same effort toward achieving positive well-being and flourishing. These exploratory findings demonstrate the importance of testing for mediation effects in order to understand the complex interrelationships between motivation and well-being. 

## 5. Conclusions

Overall, the results of this study replicated work conducted on the relationships between C and TI climates, effort, and enjoyment, albeit with a population that has not been investigated before, and introduces new insights into how climate variables correlate with flourishing and the role of mediators on that relationship. However, this study is not without limitations. Although this study examines an older group of athletes, it was not possible to explore this through a developmental lens. Future studies should employ a cross-sectional or longitudinal design to examine changes in these relationships throughout a lifespan. This study is also correlational in nature and cannot speak to causation between the variables in this study. Future work could incorporate experimental designs to assess causation or use qualitative designs to improve and strengthen our understanding of the proposed explanations for the relationships. Additionally, the correlations and effect sizes were small to moderate, indicating that there are other factors that should be considered when examining Masters athletes’ effort and enjoyment in swimming as well as flourishing in life. Finally, given the exploratory nature of this work, additional work is needed to examine other mediators between climate variables and well-being and expand our understanding by testing additional athlete outcomes.

Research has indicated that the majority of older adults are not meeting guidelines for physical activity [6]. Based on the findings from this study, fostering C and TI climates could serve as a fruitful avenue with which to promote optimal physical activity-related experiences for older adults by positively influencing their motivation to engage in a specific physical activity, namely, swimming. These climates can also set the stage for individuals’ flourishing through their influence on effort. Overall, the findings from this study add to the growing research, suggesting the importance of developing supportive environments wherein coaches and team members can emphasize striving for improvement through personal effort and create an inviting and respectful setting where all feel valued for and supported in contributing to a team. 

## Figures and Tables

**Table 1:** Descriptive statistics of all the items.

Variables	Items	Mean (*SD*)	Skewness	Kurtosis
Caring Climate		4.50 (0.58)		
(α = 0.94)	Coaches respect swimmers.	4.65 (0.73)	−2.87	10.38
	Coaches are kind to swimmers.	4.59 (0.75)	−2.62	9.17
	Coaches care about the swimmers.	4.60 (0.79)	−2.74	8.78
	Coaches try to help swimmers.	4.52 (0.85)	−2.40	6.40
	Coaches want to be acquainted with all the swimmers.	4.27 (0.98)	−1.33	1.35
	Coaches listen to the swimmers.	4.34 (0.88)	−1.54	2.67
	Coaches accept swimmers for who they are.	4.49 (0.84)	−2.05	4.99
	Swimmers are treated with respect.	4.51 (0.68)	−1.37	2.03
	Swimmers feel that they are treated fairly.	4.36 (0.77)	−1.22	1.72
	Swimmers like one another for who they are.	4.48 (0.67)	−1.09	0.86
	Swimmers feel comfortable.	4.49 (0.66)	−1.13	1.11
	Swimmers feel safe.	4.64 (0.57)	−1.10	0.00
	Swimmers feel welcome every day.	4.56 (0.67)	−1.57	2.57
Task-Involving Climate (α = 0.83)		4.14 (0.64)		
	Coaches encourage swimmers to try new skills/strokes/drills.	4.32 (0.86)	−1.36	1.81
	Swimmers of all skill/fitness levels are made to feel valued.	3.55 (1.02)	−1.33	2.15
	Coaches encourage swimmers to help each other learn.	4.27 (0.84)	−0.26	−0.53
	Coaches always emphasize trying your best.	4.37 (0.76)	−1.45	2.96
	Swimmers are rewarded and noticed when they try hard.	4.01 (0.93)	−0.86	0.31
	Sessions are focused on the continued improvement of swimming.	4.41 (0.77)	−1.60	3.46
Effort (α = 0.70)		4.26 (0.50)		
	I put a great deal of effort into swimming.	4.49 (0.58)	−0.63	−0.57
	It is important for me to perform well in terms of swimming.	4.07 (0.84)	−0.84	0.37
	I try very hard while swimming.	4.26 (0.66)	−0.76	1.07
	I do not try very hard when I swim.	4.22 (0.67)	1.13	3.24
Enjoyment		4.41 (0.49)		
(α = 0.72)	I enjoy swimming.	4.83 (0.42)	−2.80	9.64
	Swimming is fun.	4.62 (0.63)	−1.91	4.36
	I would describe swimming as very interesting.	4.06 (0.87)	−0.78	0.28
	Sometimes I think about how much I enjoy swimming.	4.22 (0.81)	−1.42	2.54
	Swimming does not hold my attention.	4.32 (0.72)	1.46	3.64
Flourishing		6.14 (0.61)		
(α = 0.90)	I lead a purposeful and meaningful life.	6.14 (0.90)	−2.11	8.11
	My social relationships are supportive and rewarding.	6.10 (0.84)	−1.44	4.21
	I am engaged and interested in my daily activities.	6.17 (0.74)	−1.15	2.86
	I actively contribute to the happiness and well-being of others.	6.05 (0.86)	−1.06	1.82
	I am competent and capable in the activities that are important to me.	6.27 (0.60)	−0.31	0.00
	I am a good person and live a good life.	6.29 (0.64)	−0.99	3.01
	I am optimistic about my future.	6.10 (0.79)	−1.13	2.19
	People respect me.	6.00 (0.89)	−1.37	2.68

Note. *n* = 294.

**Table 2:** Correlations of latent variables.

Variables	Care	Task	Effort	Enjoyment	Flourishing
Caring Climate	—				
Task-Involving Climate	0.71 **	—			
Effort	0.26 **	0.32 **	—		
Enjoyment	0.27 **	0.38 **	0.48 **	—	
Flourishing	0.14 *	0.20 **	0.26 **	0.27 **	—

Note. All correlations were positive. * *p* < 0.05; ** *p* < 0.001.

**Table 3:** Model 1 parameter estimates, standard error, and *p* values.

Hypothesized Path		Coefficient		S.E.	*p* Value
***Direct Effects***					
Caring Climate →	Flourishing	−0.007		0.140	0.954
Task-Inv. Climate →	Flourishing	0.048		0.160	0.743
Caring Climate →	Effort	−0.075		0.151	0.585
Task-Inv. Climate →	Effort	0.482 *		0.161	0.001
Caring Climate →	Enjoyment	−0.211		0.158	0.125
Task-Inv. Climate →	Enjoyment	0.645 **		0.176	0.000
Effort →	Flourishing	0.296 *		0.093	0.002
Enjoyment →	Flourishing	0.105		0.092	0.282
***Indirect Effects***		Coefficient	95% CI	S.E.	*p* value
Caring Climate → Effort → Flourishing		−0.022	−0.145−0.059	0.048	0.615
Task-Inv. Climate → Effort → Flourishing		0.143 *	0.038−0.345	0.077	0.046
Caring Climate → Enjoyment → Flourishing		−0.022	−0.099−0.031	0.034	0.478
Task-Inv. Climate → Enjoyment → Flourishing		0.068	−0.083−0.227	0.075	0.325

Note: CI = Confidence Interval; S.E. = Standard Error; * *p* < 0.05; ** *p* < 0.001; Model Fit (CFI = 0.93; TLI = 0.92; RMSEA = 0.06; SRMR = 0.05).

**Table 4:** Model 2 parameter estimates, standard error, and *p* values.

Hypothesized Path		Coefficient		S.E.	*p* Value
***Direct Effects***					
Caring Climate →	Flourishing	0.028		0.067	0.650
Caring Climate →	Effort	0.322 **		0.096	0.000
Caring Climate →	Enjoyment	0.308 *		0.119	0.007
Effort →	Flourishing	0.301 *		0.110	0.005
Enjoyment →	Flourishing	0.114		0.100	0.238
***Indirect Effects***		Coefficient	95% CI	S.E.	*p* value
Caring Climate → Effort → Flourishing		0.097 *	0.029−0.208	0.048	0.022
Caring Climate → Enjoyment → Flourishing		0.035	−0.022−0.138	0.039	0.334

Note. CI = Confidence Interval; S.E. = Standard Error. * *p* < 0.05; ** *p* < 0.001; Model fit (CFI = 0.93, TLI = 0.92, RMSEA = 0.06, SRMR = 0.06).

**Table 5:** Model 3 parameter estimates, standard error, and *p* values.

Hypothesized Path		Coefficient		S.E.	*p* Value
***Direct Effects***					
Task-Inv. Climate →	Flourishing	0.044		0.084	0.575
Task-Inv. Climate →	Effort	0.422 **		0.114	0.000
Task-Inv. Climate →	Enjoyment	0.489 **		0.117	0.000
Effort →	Flourishing	0.295 *		0.099	0.003
Enjoyment →	Flourishing	0.105		0.094	0.291
***Indirect Effects***		Coefficient	95% CI	S.E.	*p* value
Task-Inv. Climate → Effort → Flourishing		0.125 *	0.036−0.248	0.052	0.010
Task-Inv. Climate → Enjoyment → Flourishing		0.051	−0.051−0.161	0.054	0.302

Note. CI = Confidence Interval; S.E. = Standard Error. * *p* < 0.05; ** *p* < 0.001; Model fit (CFI = 0.90, TLI = 0.88, RMSEA = 0.06, SRMR = 0.06).

## Data Availability

Contact the authors about inquiries about the data.
